# Anti-inflammatory and antioxidant effects of ranolazine on primary cultured astrocytes

**DOI:** 10.1186/cc13637

**Published:** 2014-03-17

**Authors:** FB El Amrani, S Guerra, D Aguirre-Rueda, MD Mauricio, P Marchio, JM Vila, SL Vallés, F Fernández, M Aldasoro

**Affiliations:** 1 University of Valencia, Spain

## Introduction

Because of its ability to block late INa [1], ranolazine is used as an antianginal agent for the treatment of chronic angina pectoris when angina is not adequately controlled by other agents [2]. Besides its cardiovascular effects, ranolazine improves different neuronal functions, and thus its use has been proposed for the treatment of pain and epileptic disorders [3,4]. Since astrocytes are involved in neuronal inflammatory processes, and autoimmune and neurodegenerative diseases [5], we have investigated the antiinflammatory and antioxidant effects of ranolazine in primary cultured astrocytes.

## Methods

We incubated differentiated rat astrocytes in primary culture (10 days of culture) [5] for 24 hours with ranolazine (10^-5^, 10^-6^, 10^-7 ^M). We measured the protein expression levels of PPARy and Cu/Zn-SOD by western blot technique. Protective effect of ranolazine on cell viability was assayed using MTT conversion assay. Finally, to evaluate the effect of ranolazine on the IL-1β cytokine and TNFα mediators, we used the enzyme-linked immunosorbent assay technique.

## Results

Compared with control cells, treatment with ranolazine induced an increment of anti-inflammatory PPARy and reduced the proinflammatory mediators IL-1 β and TNFα in primary cultured astrocytes. Ranolazine (10^-6 ^M) also increased the expression of antioxidant protein Cu/Zn-SOD and caused a significant increase in cell viability.

## Conclusion

Ranolazine decreases inflammatory mediators IL-1 β and TNFα, and increases anti-inflammatory PPARy as well as the antioxidant Cu/Zn-SOD in astrocytes in culture. These results suggest that ranolazine could be useful as a neuroprotective drug in pathologies inducing inflammatory damage and oxidant processes.
